# Supplementary material for: Establishment of a social conditioned place preference paradigm for the study of social reward in female mice
Source: Sci Rep. 2022 Jul 4;12:11271. doi: 10.1038/s41598-022-15427-9 (PMC9253334; doi:10.1038/s41598-022-15427-9)
Supplement: Supplementary file 1 — Supplementary Information. [file 41598_2022_15427_MOESM1_ESM.pdf]

Establishment of a social conditioned place preference paradigm for the study of social reward in female mice

Zofia Harda[1], Magdalena Chrószcz[1], Klaudia Misiołek[1], Marta Klimczak[1], Łukasz Szumiec[1], Maria Kaczmarczyk-Jarosz[2], Jan Rodriguez Parkitna[1]*

Supplementary Figures

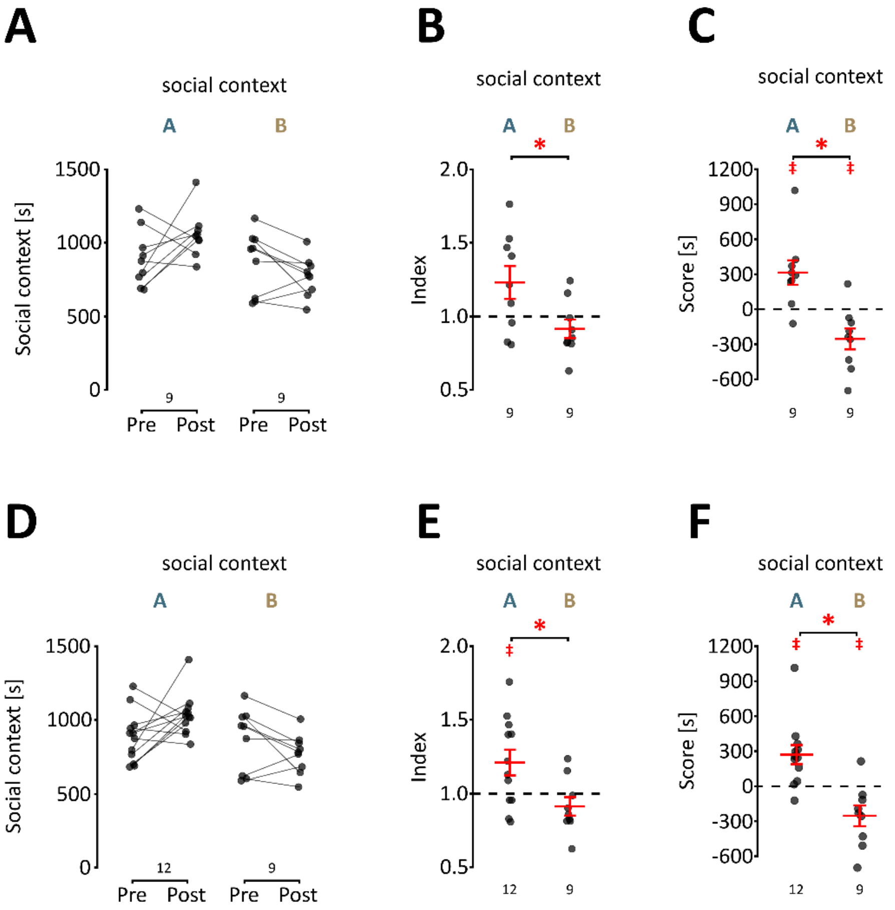

Figure S1. Acquired context preference. Panels A to C show the same data as panels H to J in Figure 2 but split between contexts, are D to F are the data before groups were "trimmed" to equal size.

(**A**) Time spent in contexts A and B during the pre- and post-test. For each animal, the times spent in the first context during pre- and post-tests are joined with a line. A significant increase from the pre- to post-test is marked with a red "*" ($p < 0.05$, two-sample t test). Group sizes are indicated below.

Statistical analysis: two-way ANOVA *context* $F_{1,16}$ = 15,85, p = 0,0264, *pre-post* $F_{1,16}$ = 0.6383 p = 0.5686; *interaction* $F_{1,16}$ = 10.96 p = 0.0282; *post hoc* Bonferroni t test context A vs. B: t_8 = 2.117 p = 0.1005 and t_8 = 1.294 p = 0.4281.

(**B**) Preference indices. The values represent the ratio between the time spent in the social context during the post-test to the corresponding time during the pre-test. The mean and s.e.m. values are shown in red. The "*" symbol represents $p < 0.05$ in two-sample t test comparison.

Comparison of A vs. B, two-sample t test, $t_{16} = 2.457$ $p = 0.0258$. One-sample t test vs. 1 (equal preference) $t_8 = 2.079$ $p = 0.0712$ and $t_8 = 1.317$ $p = 0.2244$ for contexts A and B, respectively.

(**C**) Preference scores. The values represent the difference in time spent in the social context and isolation context during the post-test. The mean and s.e.m. values are shown in red. A significant mean preference score is shown as a red "‡" ($p < 0.05$, one-sample t test vs. 0). The "*" symbol represents $p < 0.05$ in two-sample t test comparison.

Comparison of A vs. B, two-sample t test: $t_{16} = 4.130$ $p = 0.0008$. One-sample t test vs. 0 (no preference) $t_8 = 3.012$ $p = 0.0168$ and $t_8 = 2.832$ $p = 0.0221$ for contexts A and B, respectively.

(**D**, **E** and **F**) These graphs are the equivalents of B, C and D, respectively, but for mice first exposed to context B.

(D) Statistical analysis: two-way ANOVA *context* $F_{1,19} = 13.91$, $p = 0.0227$, *pre-post* $F_{1,19} = 0.4821$ $p = 0.5912$; *interaction* $F_{1,19} = 11.01$ $p = 0.0172$; *post hoc* Bonferroni t test context A vs. B: $t_{11} = 2.411$ p = 0,0524 and $t_8 = 1.366$ $p = 0.3760$.

(E) Comparison of A vs. B, two-sample t test, $t_{19} = 2.590$ $p = 0.0180$. One-sample t test vs. 1 (equal preference) $t_{11} = 2.462$ $p = 0.0316$ and $t_8 = 1.317$ $p = 0.2244$ for contexts A and B, respectively.

(F) Comparison of A vs. B, two-sample t test, $t_{19} = 4.302$ $p < 0.000$. One-sample t test vs. 0 (no preference) $t_{11} = 3.332$ $p = 0.0067$ and $t_8 = 2.832$ $p = 0.0221$ for contexts A and B, respectively.

Supplementary Tables

Table S1. Neutral conditioning context selection

	n	Context A	Context B	Excluded due to initial pref >70%	Preferred context	Preference	t test
Set 1	119	Beech 1, Block 2	Cellulose, Block 3	5	B	50.25	t_{113}=0.1189, p = 0.9056
Set 2	16	Beech 2, Block 2	Corn*, Block 3	0	B	54.25	t_{15}=1.965, p = 0.0683
Set 3	27	Beech 3, Block 2	Cellulose, Block 3	4	A	56.18	t_{22}=4.163, p = 0.0004
Set 4	10	Spruce, Block 2	Cellulose, Block 3	3	B	62.93	t_{6}=9.000, p = 0.0001

*Mice appeared to be eating the corn bedding

Table S2. The age and weight of the mice were tested for inherent bedding preference (Figure 1B)

Set	Mean age (weeks)	Age range (weeks)	Mean weight [g]	Weight range [g]
1	13.69	11.6-16.7	21.26	17.8-24.5
2	11.98	9.9-15.9	21.20	20.0-22.8
3	13.00	12.1-13.3	23.19	20.6-25.7
4	11.57	11.6-11.6	21.64	20.2-23.4

Table S3. Age and weight of animals in the sCPP experiments*

Figure	Mean age at post-test [weeks]	Age range on post-test day [weeks]	Mean weight at pre-test [g]	Weight range at pre-test [g]	Conditioning length [days]	Familiarity
1E-J	14.7	13.6-15.3	21.2	19.1-24.5	6	siblings
2B-J	14.7	13.6-15.6	21.3	19.1-24.5	6	siblings
3A-C	14.0	12.3-17.1	21.8	19.6-23.6	2	siblings
3C-D	8.7	8.4-9.0	18.8	16.7-21.1	2	siblings
4B-D	14.8	14.4-15.0	21.1	19.2-23.3	6	familiar nonsiblings
4E-G	14.6	12.6-16.1	20.4	17.8-22.9	6	unfamiliar nonsiblings

*Includes only animals shown on graphs; does not include mice with >70% initial preference or removed after trimming groups to equal size. Full dataset available: https://doi.org/10.5281/zenodo.6347482 .

Table S4. Bedding brand names and suppliers

Code	Product name	Supplier	Link
Aspen 1	ABEDD aspen animal bedding	Abedd SIA Jelgavas iela 29 Kalnciems, LV-3016 Latvia	https://www.abedd.com/
Aspen 2	Tapvei GLP aspen beeding, #HP10KG	Tapvei Estonia OÜ, Paekna küla, Kiili vald, 75408 Harjumaa, Estonia	https://tapvei.com/, https://animalab.eu/aspen-beddings
Beech 1	Trociny bukowe przesiane gat. 1	P.P.H. "WO-JAR", Kopernika 3/30, 32-100 Proszowice, Poland	-
Beech 2	Trocinka bukowa Facimiech	PPHU Natur-Drew A. Czaja, os. Kopernika 5/57, 34-100 Wadowice, Poland	-
Beech 3	LIGNOCELL ® HBK 1500-300	J. Rettenmaier and Sohne GMBH+CO KG Holzmuhle 1, D-73494 Rosenberg, Germany	https://www.jrs.eu/jrs_en/petcare/pets-dream.php
Corn	REHOFIX, Hygienic Animal Bedding	J. Rettenmaier and Sohne GMBH+CO KG Holzmuhle 1, D-73494 Rosenberg, Germany	https://www.jrs.eu/jrs_en/corn-fiber/rehofix.php
Cellulose	Biofresh Performance Bedding. 1/8' Pelleted Cellulose	ABSORPTION CORP 6960 Salashan Parkway Ferndale, WA 98248, USA	https://scottpharma.net/product/biofresh-performance-bedding/
Cotton	Podłoże bawełniane dla gryzoni	P.U i H CHABA Majora Hubala 28, 26-340 Drzewica, Poland	https://www.karusek.com.pl/produkty/chaba-podloze-bawelniane-dla-gryzoni-7l-8758
Spruce	LIGNOCELL ® Premium Hygenic Animal Bedding, Spruce Premium Scientific Bedding	Rettenmaier and Sohne GMBH+CO KG Holzmuhle 1, D-73494 Rosenberg, Germany	https://www.jrs.eu

Table S5. Gnawing blocks

Code	Name	Size [mm]	Manufacturer	Link
Block 1	SL	99 × 19 × 19	Urszula Borgiasz Zoolab, Zielona 14, 28-340 Sędziszów, Poland	http://zoolab.pl/en/enrichment-elements/
Block 2	LL	99 × 39 × 39		
Block 3	LS	49 × 39 × 39		
